# Investigating the Drought and Salinity Effect on the Redox Components of *Sulla Coronaria* (L.) Medik

**DOI:** 10.3390/antiox10071048

**Published:** 2021-06-29

**Authors:** Silvia De Rossi, Gabriele Di Marco, Laura Bruno, Angelo Gismondi, Antonella Canini

**Affiliations:** 1 Department of Biology, University of Rome “Tor Vergata”, 00133 Rome, Italy; silvia.derossi@alumni.uniroma2.eu (S.D.R.); gabriele.di.marco@uniroma2.it (G.D.M.); laura.bruno@uniroma2.it (L.B.); canini@uniroma2.it (A.C.); 2 PhD Program in Evolutionary Biology and Ecology, Department of Biology, University of Rome “Tor Vergata”, 00133 Rome, Italy

**Keywords:** abiotic stress, antioxidants, antiradical enzymes, desertification, French honeysuckle, plant defence

## Abstract

For the Mediterranean region, climate models predict an acceleration of desertification processes, thus threatening agriculture. The present work aimed to investigate the effect of drought and salinity on *Sulla coronaria* (L.) Medik., a Mediterranean forage legume, for understanding plant defence systems activated by these stressors. In detail, we focused our attention on the variations on the plant redox status. Plants were subjected to suboptimal watering and irrigation with sodium chloride (NaCl) solutions. The same salt treatment was applied for in vitro tests on seedlings. Water content did not change after treatments. Salt negatively influenced seed germination and seedling development, but it did not affect photosynthesis parameters, contrary to what was observed in adult plants. Proline concentration increased in all samples, while abscisic acid level increased exclusively in seedlings. NaCl caused accumulation of superoxide anion in plants and seedlings and hydrogen peroxide only in seedlings; nevertheless, lipid peroxidation was not detected. Total phenolics, glutathione, expression level, and activity of antioxidant enzymes were assayed, revealing a complex antiradical molecular response, depending on the type of stress and development stage. Our results confirm Sulla as a drought- and salt-tolerant species and highlight its ability to counteract oxidative stress. This evidence suggests a key role for the redox components, as signal transduction messengers, in Sulla acclimation to desertification. Finally, plants and seedlings showed different acclimation capacity to salinity, revealing a greater genomic plasticity for seedlings.

## 1. Introduction

Land degradation is an alarming global issue and climate change is expected to exacerbate it, leading in the worst cases to soil desertification. As predicted by climate models, the Mediterranean area is facing high temperature, long drought periods, and heavy rainfalls [1]. Consequently, land degradation processes are accelerated, including the phenomenon of salinization, one of the major causes of land degradation in Europe [2]. According to Salvati and Ferrara [3], one to three million hectares of land in Mediterranean countries already deal with a severe level of degradation, thus jeopardising agricultural productivity.

Plants have many and diversified strategies to defend themselves from abiotic stresses, involving responses at the morphological, physiological, and molecular level. The effectiveness of these protection systems depends on the plant’s ability to use and combine them [4]. Munns [5] demonstrated that under water and salt stress, plants share several common early responses. First, in these conditions, a physiological water deficit occurs, requiring osmotic adjustments to maintain both water uptake and cell turgor stability. Water loss by transpiration can be limited via abscisic acid (ABA)-mediated partial stomatal closure, which reduces carbon dioxide diffusion in chloroplasts and alters leaf photochemistry [6]. In addition to dehydration, a prolonged exposure to salt imposes hyper-ionic and hyper-osmotic pressures; therefore, to avoid toxic effects, plants can exclude salt or minimize its concentration in the cytoplasm, compartmentalizing it into vacuoles [7]. If stress conditions persist, plants can synthesize and accumulate intracellular compatible solutes (e.g., proline) to improve water retention. As reported by Apel and Hirt [8], stressful environmental conditions may seriously compromise the *equilibrium* between production and scavenging of reactive oxygen species (ROS), such as superoxide anion (O_2_•^−^), hydrogen peroxide (H_2_O_2_), hydroxyl radical (•OH), and singlet oxygen (^1^O_2_). Enzymatic and non-enzymatic antioxidant defence systems can counteract the detrimental effects caused by ROS accumulation on cell components. Superoxide dismutase (SOD; EC 1.15.1.1), catalase (CAT; EC 1.11.1.6), and ascorbate peroxidase (APX; EC 1.11.1.11) are the main enzymatic scavengers of ROS, while non-enzymatic antioxidants include molecules, such as ascorbate, glutathione, carotenoids, tocopherols, and flavonoids [9]. Moreover, several studies have demonstrated that abiotic factors may regulate the expression of stress-responsive genes, including those encoding for antioxidant enzymes [10,11,12,13,14].

Legumes are second, after grasses, in importance to agriculture since they represent protein sources for humans and livestock [15]. Understanding the effects of water deprivation and excess salt on legume crops is therefore crucial to preserve agriculture in adverse environmental conditions. *Sulla coronaria* (L.) Medik. (syn. *Hedysarum coronarium* L.; French honeysuckle; henceforth Sulla) is a short-lived perennial species belonging to the Fabaceae family and native to the Mediterranean basin, where it is widely used as a rainfed forage crop [16]. Indeed, this plant has great potential due to its suitability for cultivation in drought-prone and marginal environments, as testified by the ability to grow in arid and semi-arid areas, even on highly calcareous soils [17,18]. Nuti and colleagues [18] have described this legume as a good pioneer species on degraded lands. The strong root apparatus makes Sulla adequate to protect soil from erosion [19]. Moreover, its root microbiota is particularly able to improve soil fertility, fixing atmospheric nitrogen [20]. Despite these promising features, the physiological and biochemical responses of Sulla to drought and salinity conditions have been scarcely studied. According to this last evidence, the main goal of the present work was to provide novel insights into the effects of these two stressors on this species. In particular, the experiments were performed both on seedlings germinated in in vitro cultures and plants grown in soil. Photosynthetic activity and cell death induction were monitored to evaluate the consequences of the treatments on the function and viability of the plant organisms. Water retention, ABA content, and osmolyte production, strategies deemed to be critical in plant tolerance to abiotic stresses, were also investigated. Since it is known that the redox status acts as a primary sensor of stress and consequently as a signalling system capable of activating plant defence, the main aspect of this study consisted in the analysis of the plant redox components. The role of non-enzymatic antioxidants and ROS scavenger enzymes (i.e., SOD, CAT, APX) in the response of *S. coronaria* to drought and salinity was evaluated. Moreover, the activity and expression level of the antioxidant enzymes were monitored and compared. Finally, the plant response to the studied abiotic stresses was discussed.

## 2. Materials and Methods

### 2.1. Plant Material and Stress Application

Plant material was certified and provided by the Botanical Garden of Rome “Tor Vergata”. *Sulla coronaria* (L.) Medik. seeds were sterilized with 2% sodium hypochlorite for 15 min, washed three times with sterile water, and stratified at 4 °C for 72 h in the dark. Then, they were plated in 9 cm Petri dishes filled with agarized Murashige and Skoog medium (pH 5.8) and maintained under a 14 h photoperiod (18-watt fluorescent lamp) at 24 °C. For the in vitro experiments, which aimed to investigate the effect of salt treatment on seed germination and seedling development, the medium was supplemented with 100, 150, or 200 mM sodium chloride (NaCl) and the sampling was performed after 15 days of seed sowing. For the in vivo experiments, which were performed to study the resistance mechanisms of the developed plants, 15-day-old seedlings grown in non-stressing conditions were transplanted into pots, left in a greenhouse for 30 days (Supplementary Materials Figure S1), and then subjected to three different regimes of water or salt stress. In detail, the first stress consisted in a suboptimal watering (30% of field capacity, i.e., 20 mL of water every other day) for 1, 2, or 3 weeks, while the salt treatment was carried out by irrigation, every two days, with 100, 150, or 200 mM NaCl solutions for 3 weeks. Physiological parameters (i.e., biometric analysis, water content, levels of photosynthetic pigments, photosynthetic activity, and Trypan Blue staining for vitality) were measured on plant material subjected to all treatment conditions. All the other assays focused on the study of the redox state and the components of the antioxidant machinery were carried out exclusively on plants subjected to 3 weeks of suboptimal irrigation and on plants and seedlings exposed to 200 mM NaCl, since plants’ critical responses could be observed after the imposition of the maximum treatments. In vitro seedlings and in vivo plants were immediately used for analyses or placed in liquid nitrogen and stored at −80 °C. To perform the experiments, leaf material from plants corresponded to the second and third pairs of leaflets from the top of the branches, which were similar in size.

### 2.2. Biometric Analysis

After 15 days of growth in control and saline conditions, seedlings were analysed for germination, root and hypocotyl length, and cotyledon width and length, as described in [21]. In detail, germination percentage was calculated as the number of germinated seeds with respect to the total number of sown seeds × 100. Root length was measured from the tip of the root to the hypocotyl. Hypocotyl was gauged as the distance between the insertion of cotyledons on the hypocotyl and the root. For cotyledons, length was determined from the tip of the cotyledon to its insertion on hypocotyl, while the width from the upper to the lower side. All measurements, indicated in cm (for root and hypocotyl) or mm (for cotyledon), were carried out using a gauge.

### 2.3. Water Content

Water content (WC) was determined as reported by Luo et al. [22], with some modifications. At the end of each treatment, whole seedlings and whole plants were collected and immediately weighted (fresh weight, FW). Then, they were oven-dried at 37 °C for 7 days to a constant dry weight (DW). WC was calculated using the following formula: WC (%) = (FW − DW)/FW × 100.

### 2.4. Levels of Photosynthetic Pigments

Chlorophyll *a* (Chl *a*) and *b* (Chl *b*) and total carotenoid concentration was determined according to the method of Lichtenthaler [23]. Whole seedlings and leaves (100 mg) of plants were extracted in 80% acetone for 24 h, at 4 °C in the dark. After centrifugation for 5 min at 3000× *g*, supernatants were recovered and their absorbance was measured at 470, 645, and 663 nm by a UV/Vis spectrophotometer (Varian Cary 50 Bio UV-Vis, The Netherlands). Pigment levels were calculated by the application of specific equations:

(1) $$\mathrm{Chl}\;a\;(\mu g/\mathrm{mL})=12.25\;*\;{Abs}_{663}-2.79\;*\;{Abs}_{645}$$

(2) $$\mathrm{Chl}\;b\;(\mu g/\mathrm{mL})=21.50\;*\;{Abs}_{645}-5.10\;*\;{Abs}_{663}$$

(3) $$\mathrm{Carotenoids}\;\left(\mu g/\mathrm{mL}\right)=\;\frac{1000\;*\;{Abs}_{470}-\;1.82\;*\;Chl\;a\;-\;85.02\;*\;Chl\;b}{198}$$

Results were expressed as micrograms per 100 milligrams fresh weight of plant tissue (µg 100 mg^−1^ FW).

### 2.5. Photosynthetic Activity

A portable pulse amplitude modulated fluorometer (Mini-PAM; Heinz Waltz, Effeltrich, Germany), coupled with a WinControl Software (Walz GmbH, Germany), was used for detecting the electron transport rate (ETR) between photosystems II (PSII) and I (PSI) of plant samples. The formula ETR = Y × PPFD × 0.84 × 0.5 [24] was applied, where Y is the effective quantum yield of PSII photochemical reactions, PPFD is the photosynthetic photon flux density, 0.84 is a standard factor representing the fraction of incident light absorbed by photosynthetic pigments, and 0.5 is a coefficient describing the assumption that absorbed photons are equally distributed between the two photosystems. Measurements were conducted at 8 mm from the surface of cotyledons from seedlings or leaves from plants and performed with increasing PPFD values (from 0 to 1400 μmol of photons m^−2^ s^−1^).

### 2.6. Trypan Blue Staining

Cell death was monitored according to the protocol of Duan et al. [25], with some modifications. Briefly, cotyledons from seedlings and leaves from plants were immersed in 1% Trypan Blue in phosphate-buffered saline (PBS, *w*/*v*) and incubated for 45 min in the dark. After three washes with PBS, 100% ethanol was used to remove chlorophyll from samples. When completely destained, cotyledons and leaves were rehydrated by a 50% glycerol solution and scanned (Epson Perfection V700 Photo). Fiji/ImageJ software (National Institutes of Health, Bethesda, Maryland, USA) was employed for quantitation of the dark areas with respect to the total plant surface; results were reported as a percentage.

### 2.7. Proline Concentration

Free proline content was assessed following Carillo et al.’s [26] procedure. Whole seedlings and leaves of plants (50 mg) were homogenized and extracted overnight at 4 °C in 1 mL of ethanol:water solution (2:3; *v*/*v*). After centrifugation for 10 min at 14,000× *g*, 50 µL of extract were added to 100 µL of 1% ninhydrin (*w*/*v*) in 60% acetic acid (*v*/*v*) and 20% ethanol (*v*/*v*). Samples were heated at 95 °C in a block heater for 20 min, cooled at room temperature, and spun down quickly (1 min at 500× *g*). Sample absorbance was read at 520 nm by a microplate reader (Sunrise, Tecan, Austria). Proline concentration was determined using a standard curve (0–100 mg L^−1^; *R*^2^ = 0.9982) and expressed in micromoles per gram fresh weight of plant material (µmol g^−1^ FW).

### 2.8. Extraction and Quantification of ABA and Ascorbate by High-Performance Liquid Chromatography with Diode-Array Detection (HPLC-DAD)

ABA and ascorbic acid (AsA) contents were determined as reported by Nakurte et al. [27] and Gismondi et al. [28]. In detail, cotyledons from seedlings and leaves from plants were ground in liquid nitrogen and 200 mg of powdered sample were resuspended in 500 µL of 100% methanol, overnight at room temperature. After centrifugation (10 min, 16,000× *g*), supernatant was recovered and evaporated in a vacuum concentrator (Concentrator plus, Eppendorf, Germany) up to one-tenth of the initial volume. One volume of distilled water was added and then partitioned against one volume of 100% ethyl acetate. A second centrifugation (5 min, 16,000× *g*) allowed the separation of the two phases; the lower aqueous one was recovered and partitioned another time against 100% ethyl acetate. Then, samples were cleared by centrifugation (5 min, 16,000× *g*) and the upper organic phase was recovered, evaporated, and resuspended in 450 µL of 100% methanol (mobile phase). The detection of ABA and AsA was carried out using an HPLC system equipped with SPD-M20A DAD (Shimadzu, Kyoto, Japan). Identification and quantitation of the metabolites were carried out by direct comparison with increasing concentrations of pure standards (5–500 µg mL^−1^; *R*^2^ = 0.9989 for ABA and *R*^2^ = 0.9974 for AsA) (Sigma-Aldrich, St. Louis, Missouri, USA), based on the retention time, absorbance spectrum, and chromatographic peak area. The analysis was performed using a Phenomenex Luna 3u C18 (2) (3 µm × 4.6 mm × 150 mm) column, formic acid 1% (phase A) and methanol (phase B) as solvents, and an elution gradient set as follows: *t*0 min (A 85%, B 15%); *t*20 min (A 65%, B 35%); *t*55 min (A 10%, B 90%); *t*68 min (A 85%, B 15%); *t*70 min (end run) [28].

### 2.9. Reactive Oxygen Species Detection

Superoxide anion presence was monitored by staining with nitro-blue tetrazolium (NBT) according to Wohlgemuth et al. [29], with modifications. Cotyledons from seedlings and leaves from plants were immersed in 20 mM phosphate buffer (pH 6.1), containing 2 mM NBT, and incubated overnight in the dark. Reaction was stopped by moving samples in distilled water and then in 50% glycerol solution. Fiji/ImageJ software (National Institutes of Health, Bethesda, Maryland, USA) permitted analysis of high-resolution images acquired by scanning (Epson Perfection V700 Photo). Superoxide anion was visualized as a dark-blue signal within plant tissues, due to formazan generation, and quantified as a percentage of the stained area with respect to the total plant surface. Hydrogen peroxide present in plant tissues was detected using 2′,7′-dichlorofluorescein diacetate (DCFH-DA) probe, as described by Rodríguez-Serrano et al. [30]. Cotyledons from seedlings and leaves from plants were incubated in the dark for 30 min at 37 °C, with 10 mM tris(hydroxymethyl)aminomethane hydrochloride (Tris-HCl, pH 7.5) buffer enriched by 25 µM DCFH-DA. Then, samples were washed three times with the same buffer, placed on a microscope slide, and observed by a FluoView 1000 Olympus IX81 inverted Confocal Laser Scanning Microscope (Shinjuku-ku, Tokyo, Japan) equipped with a fluorescein isothiocyanate (FITC) filter (green). The acquired images were analysed by Fiji/ImageJ software (National Institutes of Health, Bethesda, Maryland, USA). Positive and negative controls were performed by exposing samples to 5 mM H_2_O_2_ and 1 mM of ascorbate, respectively, for 1 h in agitation at 37 °C. Results were expressed as a percentage of intracellular fluorescence areas compared to the total plant surface.

### 2.10. Lipid Peroxidation

The amount of malondialdehyde (MDA), a by-product of lipid peroxidation, was measured using the thiobarbituric acid (TBA) method [31]. Whole seedlings and leaves of plants were ground in liquid nitrogen and 80 mg were homogenized in 0.1% trichloroacetic acid (TCA). Samples were centrifuged at 12,000× *g* for 10 min and 400 µL of the supernatant were mixed to 1 mL of 0.5% TBA (*w*/*v*) in 20% TCA (*w*/*v*). After incubation at 95 °C for 30 min, the mixture was quickly cooled in ice. The absorbance was recorded at 532 nm by a microplate reader (Sunrise, Tecan, Austria) and corrected for non-specific absorbance at 600 nm. The MDA concentration was calculated using an extinction coefficient of 155 mM^−1^ cm^−1^ and expressed as nanomoles per gram of fresh weight of plant material (nmol g^−1^ FW). 

### 2.11. Total Phenolic Content

Total phenolics were detected by the Folin-Ciocalteu spectrophotometric assay, according to the guidelines reported in Gismondi et al. [32] with slight modifications. Frozen samples were ground to fine powder with a mortar and pestle in liquid nitrogen and 100 mg were dissolved in methanol for 24 h at 4 °C. Solvent was removed by a vacuum concentrator (Concentrator plus, Eppendorf, Germany) and samples were resuspended in 50 μL of bidistilled water; then, 500 μL of Folin-Ciocalteu reagent (1:10, *v*/*v*) and 450 μL of 0.7 M sodium carbonate (Na_2_CO_3_) were added. The mixture was incubated for 60 min in the dark and the absorbance was read at 760 nm by a microplate reader (Sunrise, Tecan, Austria). A standard curve (0–30 mg L^−1^; *R*^2^ = 0.994), prepared with gallic acid (GA; Sigma-Aldrich, USA), permitted the quantitation of total phenolics, expressed in micrograms of GA equivalents per 100 milligrams fresh weight of plant material (μg GAE 100 mg^−1^ FW). 

### 2.12. Glutathione Quantitation

For glutathione analysis, the protocol of Sahoo et al. [33] was applied. Powdered samples (50 mg) were homogenized in 500 µL of 6% metaphosphoric acid, containing 1 mM ethylenediaminetetraacetic acid (EDTA). After centrifugation at 11,500× *g* for 15 min at 4 °C, supernatants were recovered and 100 µL were added to 250 µL of 0.5 M potassium phosphate buffer (pH 7.5). Then, 25 µL of 10 mM 5,5′-dithio-bis-(2-nitrobenzoic acid) (DTNB), 50 µL of 10 mM bovine serum albumin (BSA), and 25 µL of 0.5 mM β-nicotinamide adenine dinucleotide, reduced disodium salt (NADH) were also added to the sample. After incubation at 37 °C for 15 min, the absorbance was read at 412 nm for total glutathione quantitation. After this step, reduced glutathione (GSH) was removed from the same sample by addition of 2-vinylpyridine (1:10), for 1 h at 25 °C. Then, 25 µL of extract were mixed with 150 µL reaction buffer (100 mM potassium phosphate buffer; 5 mM EDTA; pH 7.5), 100 μL of diluted yeast glutathione reductase (20 U/ml), and 100 μL of 10 mM DTNB. The reaction was triggered by 100 μL of 2.5 mM β-nicotinamide adenine dinucleotide 2′-phosphate reduced tetrasodium salt hydrate (NADPH) and the absorption was monitored at 412 nm, to obtain the oxidized glutathione concentration (GSSG). Quantitation was performed by extrapolation from a standard curve obtained using increasing amounts of glutathione. The GSH level was estimated by subtracting GSSG from total glutathione. Results were reported as a concentration (µM 50 mg^−1^ FW).

### 2.13. Antioxidant Enzyme Extraction and Activity Assays

Antioxidant enzymes were extracted as described by Di Marco et al. [21], with modifications. All operations were performed at 4 °C. Whole seedlings and leaves of plants were ground to fine powder in liquid nitrogen. For SOD activity measurement, 50 mg of plant material were homogenized with 500 µL of 50 mM potassium phosphate buffer (pH 7.8), containing 2% (*w*/*v*) polyvinylpolypyrrolidone (PVPP), 1 mM EDTA, and 0.1% Triton X-100. For CAT and APX assays, sample homogenization was performed with 500 µL of 100 mM sodium phosphate buffer (pH 7.0), containing 2% (*w*/*v*) PVPP, 1 mM EDTA, 1% Triton X-100, 10% glycerol, and 1X protease inhibitor cocktail; exclusively for APX extraction, 5 mM ascorbate was also added to the buffer. Homogenates were centrifuged at 20,000× *g* for 20 min and supernatants were used for analyses. Protein concentration was measured by the spectrophotometric method of Bradford [34], using bovine serum albumin as a standard.

SOD activity was determined by its ability to inhibit photochemical reduction of the yellow NBT to blue formazan [35]. The reaction mixture contained 1 mM EDTA, 75 µM NBT, 10 mM L-methionine, and 10 µM riboflavin (the source of O_2_•^−^ in the presence of light) in 50 mM potassium phosphate buffer (pH 7.8). Finally, 50 µg of protein sample were added to the mixture and illuminated for 15 min. The absorbance was read at 560 nm with a microplate spectrophotometer (Sunrise, Tecan, Austria). One unit of SOD activity was defined as the amount of enzyme that produced 50% of NBT photoreduction; results were reported as a unit per microgram of plant protein (U µg^−1^ protein).

CAT activity was assayed as indicated in Iwase et al. [36]. A calibration curve was arranged (0–100 U; *R*^2^ = 0.9839) with known CAT units, derived from appropriate dilutions of pure CAT (Sigma-Aldrich, USA). In total, 100 µL of each catalase standard solution or sample were placed in a tube with 1% Triton X-100 (100 µL) and 30% H_2_O_2_ (100 µL; final concentration 3.26 M). Mixtures were shaken and incubated for 15 min at room temperature. The O_2_-forming foam that remained constant was measured in a graduated test tube, where the reaction happened. Results were expressed as a unit per microgram of plant protein (U µg^−1^ protein).

APX activity was detected following Mittler and Zilinskas’ [37] procedure, with modifications. Polyacrylamide gel electrophoresis was conducted under denaturing conditions (SDS-PAGE). Polyacrylamide gels (10% resolving and 4% stacking) were pre-run for 30 min at 4 °C to allow the ascorbate (2 mM) present in the carrier buffer to enter the gels. Samples were loaded and electrophoresis separation was performed for 6 h at 4 °C. Sodium dodecyl sulphate (SDS) removal and protein renaturation were obtained by washing gels with 2.5% Triton X-100 solution, for 1 h 20 min. Then, gels were immersed for 30 min in an equilibration buffer (50 mM sodium phosphate buffer pH 7.0, containing 2 mM ascorbate) and incubated for 20 min with 50 mM sodium phosphate buffer containing 4 mM ascorbate and 2 mM H_2_O_2_. After a quick wash in water, gels were stained with 50 mM sodium phosphate buffer (pH 7.8), containing 28 mM tetramethylethylenediamine (TEMED) and 2.45 mM NBT. Gel images were acquired using a VersaDoc (Bio-Rad) instrument and spot quantitation was performed with Quantity One software (Bio-Rad).

### 2.14. Quantitative Real-Time PCR (qRT-PCR)

For qRT-PCR investigations, total RNA was extracted from 50 mg of whole seedlings or leaves from plants. To do it, plant material was incubated for 1 h, at 37 °C, with 500 μL of lysis buffer (1.5 M Tris-HCl pH 7.5, 5 mM NaCl, 0.5 M EDTA, 10% SDS, 5% polyvinylpyrrolidone, 5 mg/ml Proteinase K). Then, 50 μL of 3 M sodium acetate (pH 4.0), 50 μL of 0.5 M EDTA (pH 8.0), and a volume of phenol/chloroform (pH 4.0) were added. Solution was mixed by inversion and centrifuged for 5 min at 15,000× *g*. Supernatant was collected, and the phenol/chloroform step was repeated two more times. Finally, a volume of 2.5% cetyltrimethylammonium bromide (CTAB) solution was added and the mixture was kept in agitation for 1 h. After centrifugation, pellet was discarded, and a volume of chloroform was added. After centrifugation, the upper phase was collected, and 0.7 volumes of isopropanol were added. Sample was placed overnight at −80 °C and then centrifuged for 30 min at 4 °C at 12,000× *g*. All liquid was removed, and pellet resuspended in RNase-free water. RNA quantity and quality were analysed spectrophotometrically using a Nanodrop 1000 Spectrophotometer (Thermo Scientific, Wilmington, Delaware, USA). For cDNA synthesis, 2.5 μg of RNA were denatured at 65 °C for 2 min and retrotranscribed using 10 pmol dNPTs, 200 U M-MLV reverse transcriptase (Promega, Milan, Italy), 1X reverse transcriptase buffer, 10 mM 1,4-dithiothreitol (DTT), 0.5 μg random examers, and 20 U RNase inhibitor. Sample was then incubated at 37 °C for 90 min. Based on Novelli et al. [38], each qRT-PCR reaction was performed by mixing 25 ng of cDNA, 1X SYBR Green PCR Master Mix (Perkin-Elmer Applied Biosystems, Waltham, MA, USA), and 5 pmol of forward and reverse primer (Supplementary Materials Table S1). Amplifications were performed using the StepOnePlus Real-Time PCR System (Perkin-Elmer Applied Biosystems) set as reported: (i) initial denaturation at 95 °C for 4 min; (ii) 45 cycles of denaturation at 95 °C for 1 min, primer annealing at 58 °C for 1 min, and extension at 72 °C for 1 min; (iii) production of dissociation curve, from 50 to 95 °C (rate: 0.3 °C every 15 s). For each analysed gene, the relative amount of transcript was measured using the 2^−ΔΔCt^ formula [39], where the threshold cycle (Ct) of the target gene was firstly normalized with the internal reference gene (Actin, ΔCt; also, GAPDH was used as an internal loading control and results were comparable) and then for the respective value obtained in the control sample (ΔΔCt), which was considered as a unit. In this study, the validation of 2^−ΔΔCt^ method was carried out by ΔCt variation analysis at different template concentrations, as widely described in Livak and Schmittgen [39]. 

### 2.15. Statistical Analysis

All experiments were repeated in quadruplicate. Results are reported as means ± standard error (S.E.) of at least three independent measurements. Data were subjected to one-way analysis of variance (ANOVA) and the mean differences were compared by the post-hoc lowest standard deviations (LSD) test, using PAST software (*p* values: * *p* <0.05; ** *p* <0.01; *** *p* <0.001).

## 3. Results

### 3.1. Effects of NaCl on Seed Germination

**Figure 1:**
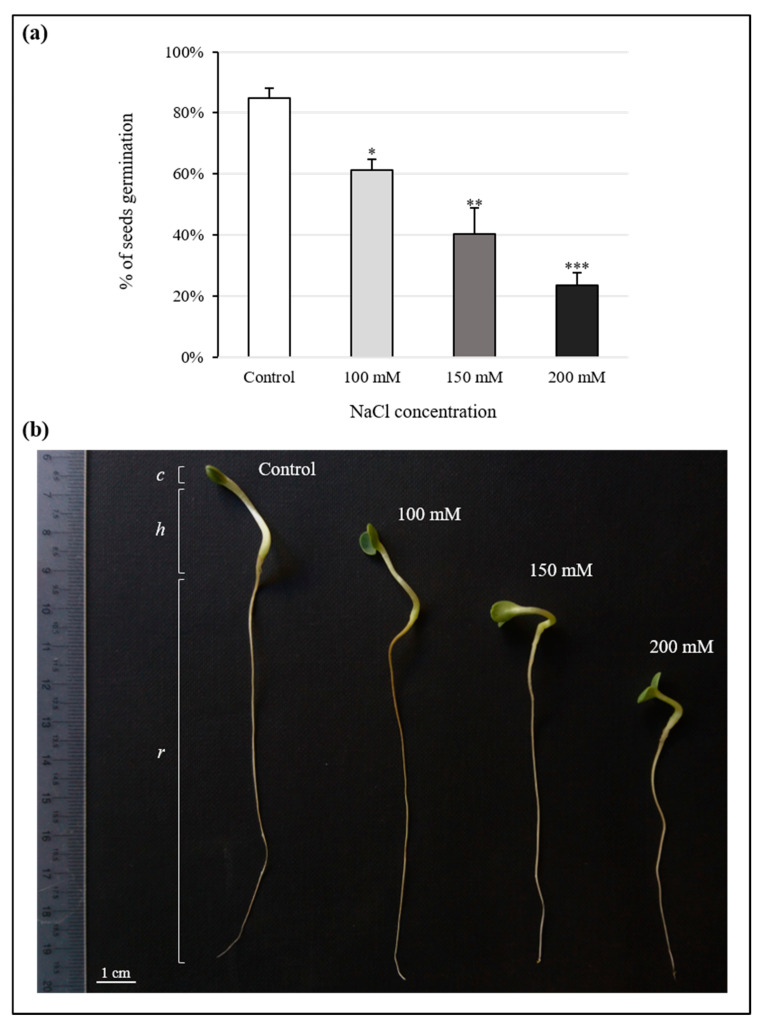
Stress effect on seed germination and seedling growth. (**a**) Effect of salt treatments (100, 150, and 200 mM NaCl) on the germination of *Sulla coronaria* seeds in in vitro conditions, after 15 days of sowing. The total number of seeds sown represents the 100%. Values, reported as percentage, are means ± S.E. based on four independent experiments (*n* = 4). Asterisks denote significant differences from control samples (one-way ANOVA/LSD test; *** *p* < 0.001, ** *p* < 0.01, * *p* < 0.05); (**b**) Morphological changes in seedling development of *Sulla coronaria* after 15 days of treatment with different NaCl concentrations (100, 150, and 200 mM); cotyledons, hypocotyl, and root are indicated by letters *c*, *h*, and *r*, respectively. The bar indicates 1 cm.

Salt stress inhibited the germination of *S. coronaria* seeds in in vitro conditions; this effect was enhanced at increasing concentrations of NaCl (Figure 1a). In particular, the treatments with 100, 150, and 200 mM NaCl significantly reduced the germination by 39, 60, and 76%, respectively, compared to the control. 

Figure 1b and Table 1 show the effect of NaCl on seedling morphology. None of the treatments produced significant changes in cotyledons. Exposures to 150 and 200 mM NaCl shortened the hypocotyl length by 35 and 57%, in that order. Root length was also affected by the stressor, decreasing by 47 (150 mM) and 51% (200 mM). 

### 3.2. Evaluation of Plant Physiological State after Exposure to Abiotic Stressors

To evaluate the physiological conditions of Sulla samples, water content, cell viability, and photosynthetic parameters were measured. WC did not show any significant variation either in seedlings or in plants, with respect to the controls, during drought and salt treatment (Supplementary Materials Figure S2).

Likewise, cell death, evaluated by Trypan Blue staining, revealed no significance difference among treatments and controls, settling on very low percentages (<1%; Supplementary Materials Figure S3).

Photosynthetic pigmentation appeared altered depending on the applied stress type (i.e., drought or salinity) and the developmental stage of *S. coronaria* (i.e., plants or seedlings), as reported in Table 2. In plants subjected to water stress, an increase in Chl *a* and carotenoid content was registered after 2 weeks of treatment; then, all pigments declined by about 50%, with respect to the controls, after the third week. In plants, salt stress caused a significant reduction of photosynthetic pigments (about 60% compared to the control) at all NaCl concentrations. By contrast, in seedlings, an enhancement in pigment content was observed. In particular, the highest treatment (200 mM NaCl) determined, compared to the controls, an increase in Chl *a*, Chl *b*, and carotenoids by 126, 48, and 77%, respectively. 

**Figure 2:**
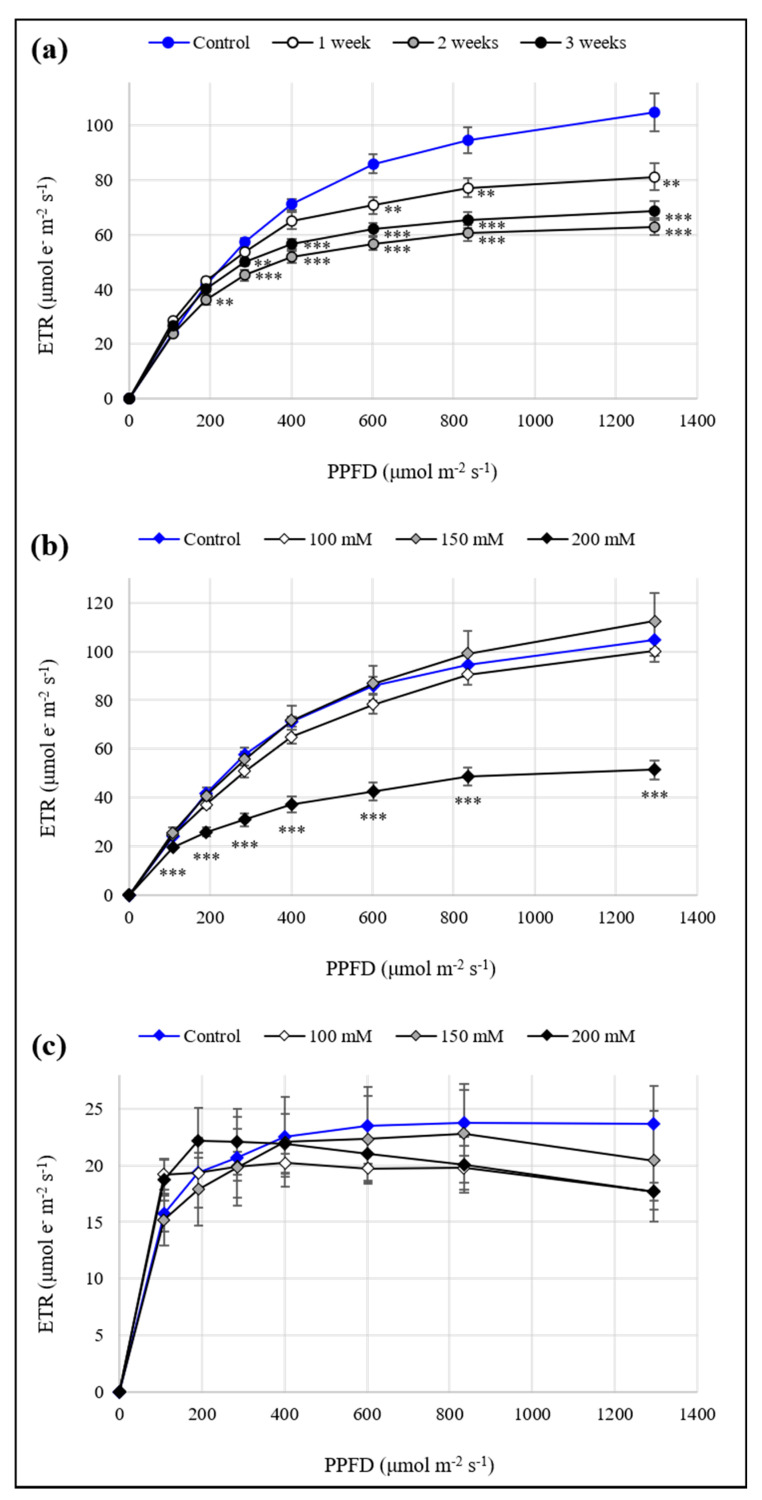
Effect of various levels of drought and salinity on the electron transport rate (ETR) in *Sulla coronaria*: (**a**) leaves of plants subjected to suboptimal watering for 1, 2, or 3 weeks; (**b**) leaves of plants treated with 100, 150, or 200 mM NaCl solutions for 3 weeks; (**c**) cotyledons of seedlings grown for 15 days in in vitro conditions on agarized culture medium supplemented with 100, 150, or 200 mM NaCl. Values shown are means ± S.E. of three independent experiments (*n* = 3). The asterisks below the error bars indicate statistically different means (one-way ANOVA/LSD test) at *** *p* < 0.001, ** *p* < 0.01, * *p* < 0.05.

The measurements of ETR allowed us to evaluate the efficiency of the photosystems. All drought treatments caused a reduction in the photosynthetic activity of plants (Figure 2a). Under salt stress, plants did not show significant differences, except at the highest concentration of NaCl, where a considerable reduction in the photosynthetic efficiency was observed (Figure 2b). Finally, in seedlings, salinity did not affect ETR values, which were in all cases like the control (Figure 2c).

Since critical responses were observed after the imposition of the maximum treatments, we decided to focus our attention exclusively on plants subjected to 3 weeks of suboptimal irrigation and on plants and seedlings exposed to 200 mM NaCl.

### 3.3. Proline and ABA Contribution to Osmotic Adjustment

**Figure 3:**
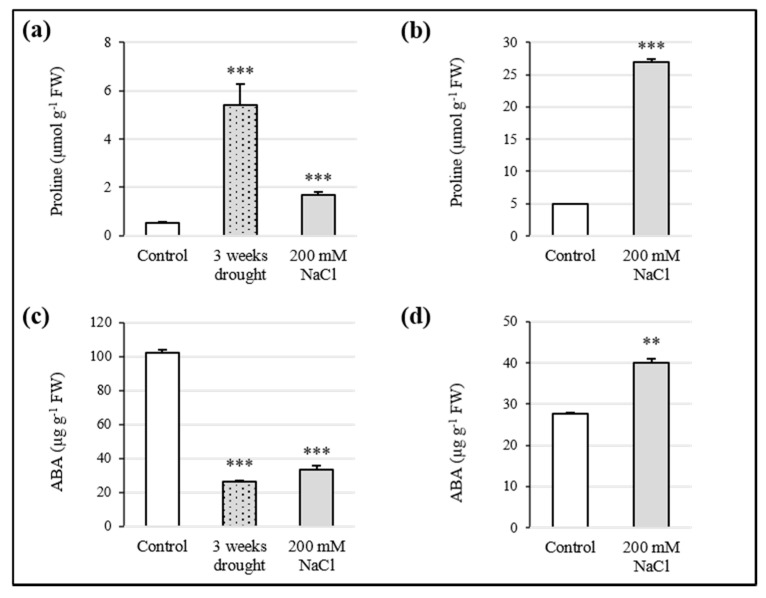
Proline and ABA content in *Sulla coronaria*. Proline concentration in leaves of plants subjected to drought and salinity (**a**) and in whole seedlings grown in 200 mM NaCl (**b**). ABA levels in leaves of plants after exposure to water and high salt stress (**c**) and in whole seedlings after imposition of saline conditions (**d**). All data are means ± S.E. of three different measurements (*n* = 3). Bars with asterisks are significantly different with respect to the control (one-way ANOVA/LSD test) at * *p* < 0.05, ** *p* < 0.01, ***p < 0.001.

The determination of proline was carried out by the spectrophotometric method. Proline concentration increased significantly due to both water and high salt stress. Compared to the control, proline level rose 10-fold in plants subjected to 3 weeks of drought and 3-fold in leaves of organisms exposed to 200 mM NaCl (Figure 3a). In seedlings, proline was found to be 5-fold higher than control, after 15 days of growth in agarized medium supplemented with 200 mM NaCl (Figure 3b).

All samples were subjected to HPLC-DAD analysis for the quantitation of ABA. ABA content was strongly affected by the water deficit and high salt. In the first case, a decrease of 75% in Sulla leaves was observed, while 200 mM NaCl caused a reduction of the hormone level equal to 67%, with respect to the control (Figure 3c). On the other hand, in seedlings, salinity caused an enhancement of ABA by 45% (Figure 3d).

### 3.4. ROS Detection and Cell Membrane Integrity 

**Figure 4:**
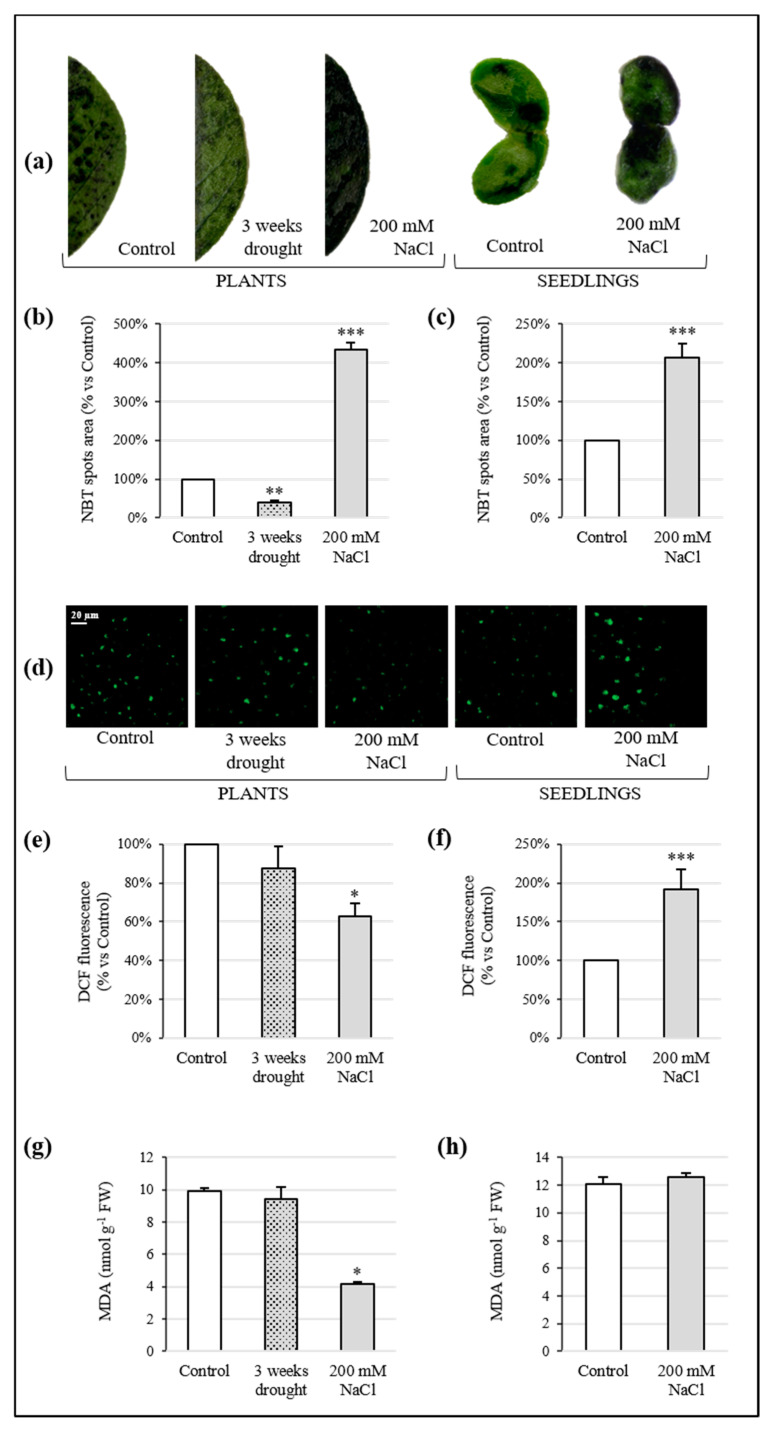
Presence of ROS in tissues of *Sulla coronaria* exposed to drought and salinity and their effects on membrane integrity. (**a**) In vivo localization of O_2_•^−^ in leaves of plants and in cotyledons of seedlings by histochemical staining with NBT. Superoxide quantification in leaves from plants (**b**) and in cotyledons from seedlings (**c**). In vivo localization of H_2_O_2_ in leaves of plants and in cotyledons of seedlings by histochemical staining with DCFH-DA; the bar indicates 20 µm (**d**). Hydrogen peroxide concentration in leaves of plants (**e**) and in cotyledons of seedlings (**f**). Changes in lipid peroxidation, in terms of MDA content, in leaves of plants exposed to drought and salinity (**g**) and in whole seedlings (**h**) grown in saline conditions. All values are means ± S.E. of three different replicates (*n* = 3). Asterisks denote significant differences from control samples (one-way ANOVA/LSD test; *** *p* < 0.001, ** *p* < 0.01, * *p* < 0.05).

Superoxide anion and H_2_O_2_ were detected by histochemical staining based on reagents able to reveal the presence of these radicals. The concentration of O_2_•^−^ in leaves of Sulla plants was altered by drought and salinity. In detail, superoxide anion decreased by 59% after 3 weeks of water deficiency, while it increased to over 4-fold when high salt treatment was applied (Figure 4a,b). Likewise, in seedlings exposed to 200 mM NaCl, O_2_•^−^ production was 2-fold higher than the control (Figure 4a,c).

The hydrogen peroxide content in plants did not show significant variation in comparison to the control after drought imposition. Otherwise, this compound significantly lessened in response to the 200 mM NaCl treatment, decreasing by 37% (Figure 4d,e). An increase of almost 2-fold in H_2_O_2_ concentration was observed in high salt-treated seedlings, with respect to the control (Figure 4d,f). Negative and positive controls were performed to check the effectiveness of the assay (Supplementary Materials Figure S4).

The levels of lipid peroxidation, assessed as MDA content, are shown in Figure 4g,h. With respect to the control, drought stress did not affect MDA concentration in Sulla plants, whereas salinity decreased the MDA level by 58%. By contrast, NaCl did not cause significant change in the seedlings for this parameter.

### 3.5. Regulation of Non-Enzymatic Antioxidant Defence

The influence of water deprivation and high salt exposure on the content of phenolic compounds, glutathione, and AsA was investigated. Total phenolics, measured by the spectrophotometric approach, did not change significantly in plants subjected to drought stress, with respect to the control (Table 3). After high salt treatment, Sulla leaves presented a phenolic content lower than the control, decreasing of 58%. By contrast, salinity caused a strong enhancement of simple phenolics (+133%) in treated seedlings.

In Table 3, the quantitation of total glutathione in Sulla samples is reported. Values comparable to the control were registered in plants exposed to drought and salinity. On the contrary, in seedlings, the glutathione concentration decreased by 38% with respect to the control. The GSSG/GSH ratio was estimated for each sample, showing an increase as a consequence of the stress imposition.

Although the AsA level slightly varied between control and treated samples, the HPLC-DAD results were not significant (Table 2).

### 3.6. Analysis of Antioxidant Enzyme Activity

**Figure 5:**
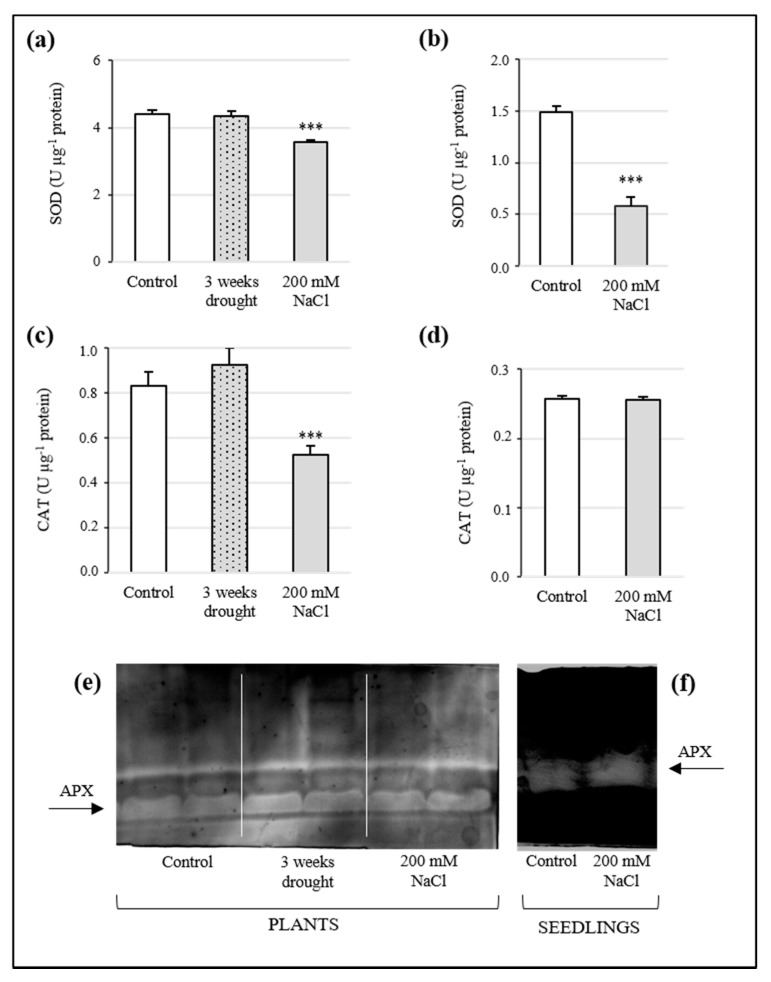
Activity of SOD, CAT, and APX in the leaves of plants (**a**,**c**,**e**) and whole seedlings (**b**,**d**,**f**) of *Sulla coronaria* after imposition of water and high salt stress. Data are reported as means ± S.E. of three independent experiments (*n* = 3). The asterisks indicate the degree of significance with respect to the control (one-way ANOVA/LSD test): *** *p* < 0.001, ** *p* < 0.01, * *p* < 0.05.

The antioxidant enzyme activities in plants and seedlings of Sulla are shown in Figure 5. Under water stress conditions, SOD activity in plants appeared similar to the control, settling on 4.34 ± 0.16 and 4.42 ± 0.11 U µg^−1^ protein, respectively (Figure 5a). By contrast, high salt-treated plants registered a remarkable decrease of SOD function by 19% (3.56 ± 0.08 U µg^−1^ protein). In the same way, as reported in Figure 5b, in seedlings, 200 mM NaCl caused a significant reduction of 61% in SOD activity (0.58 ± 0.08 U µg^−1^ protein), with respect to the controls (1.49 ± 0.06 U µg^−1^ protein).

The bioactivity of CAT was investigated, and the results are shown in Figure 5c,d. The enzyme activity was not affected by drought (0.92 U ± 0.08 µg^−1^ protein), remaining comparable to the control (0.83 ± 0.06 U µg^−1^ protein), while in high salt-treated plants, CAT function decreased by 37% (0.52 ± 0.04 U µg^−1^ protein). Seedlings subjected to NaCl stress did not show remarkable variations with respect to the control.

The APX activity detected in Sulla plants subjected to both stressors did not register significant variations, compared to the control (Figure 5e). A non-significant increase in APX activity was recorded in seedlings exposed to 200 mM NaCl, with respect to untreated samples (Figure 5f).

### 3.7. Expression of Genes Encoding for Antioxidant Enzymes

**Figure 6:**
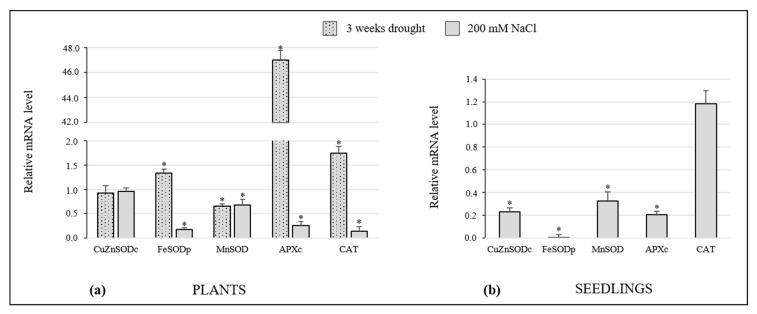
Effect of water and high salt stress on gene expression of SOD isoforms (cytosolic CuZnSOD—CuZnSODc; plastidial FeSOD—FeSODp; mitochondrial MnSOD-MnSOD), cytosolic APXc and CAT in the leaves of plants (**a**) and whole seedlings (**b**) of *Sulla coronaria*. The mRNA levels were normalized with respect to actin and expressed in arbitrary units, with respect to the respective control (considered as unit, 1). Data are means ± S.E. of three different experiments (*n* = 3). Bars with asterisks are significantly different with respect to control values (one-way ANOVA/LSD test) at * *p* < 0.05.

Gene expression analysis of Sulla plants and seedlings was performed by measuring the transcript levels of enzymes involved in ROS metabolism, with respect to the control (considered as unit, 1) (Figure 6).

In the present study, the amount of cytosolic CuZnSOD (CuZnSODc), plastidial FeSOD (FeSODp), and mitochondrial MnSOD (MnSOD) mRNAs was investigated. CuZnSODc transcript remained constant in Sulla plants subjected to drought and salinity, showing comparable expression levels with the control systems. On the other hand, its expression was significantly suppressed in high salt-treated seedlings. FeSODp expression was induced in plants after 3 weeks of drought, whereas a notable decrease was observed in the leaves of plants exposed to 200 mM NaCl. NaCl strongly inhibited the expression of this gene in seedlings, too. Finally, MnSOD mRNA was inhibited in drought- and high salt-stressed plants, presenting comparable values between them. This SOD isoform was also downregulated in seedlings treated with 200 mM NaCl.

The transcription levels of two enzymes involved in H_2_O_2_ scavenging, cytosolic APX (APXc) and peroxisomal CAT, were monitored. The first one, involved in the Asada–Halliwell pathway [40], detoxifies H_2_O_2_ oxidizing ascorbate, whereas the second decomposes H_2_O_2_ in water and oxygen [41]. The APXc mRNA level was significantly higher in drought-stressed plants compared to control samples. On the contrary, salt stress, both in plants and seedlings, induced a strong reduction in the transcript amount of this enzyme. 

The trend of CAT gene expression in plants was similar to that of FeSODp, whereas in seedlings grown under high salt conditions, CAT mRNA showed a value comparable to the control.

## 4. Discussion

Nowadays, drought and salinization are the major environmental threats to agriculture, as they limit crop productivity in arid and semi-arid regions. *S. coronaria* is a plant widely cultivated in the Mediterranean basin because of the high quality of its forage. Sulas and colleagues [16] documented the capacity of Sulla to survive in drought-prone and marginal areas, making it a promising model organism in a climate-changing world and a good candidate for drought tolerance studies. However, the ability of this species to deal with salinity is still debated and the present research attempted to investigate this issue, focusing our attention on the role of the redox components and the variation of tissue oxidative status.

Salt treatments, at all selected concentrations (100, 150, and 200 mM), negatively influenced both seed germination and shoot development, limiting hypocotyl and root elongation, in a dose-dependent manner. It is known that the presence of salt in culture media can reduce water uptake and/or cause toxic effects on seeds [42]. This might explain the germination percentages observed in our work. Regarding seedling growth, similar evidence on Sulla was reported by Kaddour et al. [43], suggesting a high sensitivity to similar concentrations of salt for this species. Curiously, saline stress did not seem to affect the cotyledons, probably a plant strategy to preserve the efficiency of the photosynthetic apparatus and to compensate for the detrimental consequences of salinity. The spectrophotometric quantitation of photosynthetic pigments and the detection of photosynthesis efficiency by a Mini-PAM supported this hypothesis. In fact, in seedlings, the content of Chl *a*, Chl *b*, and carotenoids increased, or remained unchanged, and the ETR curves showed a good electron transport mechanism, regardless of the NaCl concentration. Salinity, as well as drought, usually imposes a decrease in the chlorophyll and carotenoid content. Thus, the ability of a plant to maintain steady levels of these pigments under stressful conditions is considered indicative of the stress tolerance [4,44]. Kaddour et al. [43] reported that photosynthetic pigments in *S. coronaria* remained unaffected after exposure to high salt concentrations (e.g., 200 mM). Generally, osmotic stress conditions, such as those caused by salinity and drought, can lead to a reduction of the water content, threatening physiological and structural plant homeostasis [6]. The conservation of the water status we observed in seedlings subjected to salt stress might explain the good functioning of the photosynthetic machinery. The same phenomenon was also demonstrated in Sulla leaves of adult plants, as a result of both water and salt stress. Likely, Sulla is able to accumulate an osmoprotectant and thus avoid loss of turgor. Unlike seedlings, photosynthetic pigments and ETR were negatively affected in adult plants subjected to three different regimes of salt and water stress, which is suboptimal watering for 1, 2, or 3 weeks and irrigation with 100, 150, or 200 mM NaCl solutions for 3 weeks, respectively. In particular, salinity heavily decreased pigment content. In addition, less efficient photosynthesis was recorded at the highest NaCl concentration. During drought stress, negative effects on chlorophylls and carotenoids were observed exclusively after three weeks of treatment, while photosynthesis was always less efficient than the control. Genome plasticity and resilience of young cells/tissues probably represent the key factors underlying the different mechanisms of the plant response observed in seedlings and plants of Sulla. Seedlings, germinating and growing in a stressful condition, appeared prone to developing the best acclimation strategy to deal with osmotic and toxic effects induced by the stressors. By contrast, mature plants, experiencing stress later (that is, when the development is complete), showed more limited and less available acclimation schemes. Indeed, seed germination and seedling development are critical phases for plant establishment [45], and probably, at these stages, the environmental stress response has greater potential. The preliminary data reported above highlighted that Sulla triggered its defence strategy primarily after the imposition of the maximum treatments. Therefore, we decided to focus the next experiments on plants subjected to 3 weeks of suboptimal irrigation and on plants and seedlings exposed to 200 mM NaCl.

The conservation of water content in Sulla after stress exposure was a surprising outcome. For this reason, the presence of proline and the involvement of ABA were investigated. In the presence of abiotic stresses, the synthesis of non-cytotoxic organic solutes is a common plant response to achieve osmotic adjustments [15]. Proline is one of the main osmolytes involved in plant cell osmoregulation. In addition, it has also been considered a non-enzymatic antioxidant capable of scavenging ROS and a protective agent for protein integrity and enzyme activity, enabling plants to tolerate environmental stressors [46]. Our results, both in plants and seedlings, suggested for proline an active role in regulating Sulla water balance, increasing intracellular molarity, and capturing water by osmosis. This evidence, in line with data on water content, also indicated a possible implication of this molecule in maintaining redox *equilibrium*. Munns and Tester [47] affirmed that the synthesis of compatible solutes occurs with high energy costs, which potentially affect the plant growth, as we observed de facto in seedlings treated with 200 mM NaCl. The unaltered functioning of the photosynthetic machinery detected in the same samples might be explained by the accumulation of carotenoids able to protect the photosynthetic apparatus against stress-induced ROS [48]. ABA is a stress-responsive hormone and both drought and salinity can trigger its de novo synthesis in roots. Then, this molecule is transported mainly to the leaves, where it promotes stomatal closure, resulting in regulation of the transpiration rate and preservation of cell turgor [6,42,49]. The detection of ABA in Sulla samples by HPLC-DAD revealed a significant increase of this molecule in seedlings, and, unexpectedly, a reduction in leaves of adult plants, at the end of water and high salt stress treatments. For seedlings, the involvement of ABA as an additional protagonist in the osmotic adjustment was clearly assumed. However, ABA biosynthesis induced by abiotic stress still needs to be understood. ABA-independent response pathways have been documented in plants exposed to such a type of environmental *stimulus* [50]. Thus, the reduction of ABA in Sulla plants may suggest that it was not directly involved in the defence mechanism. Its low levels could be justified by the absence of a water deficit in the samples, as the production of this hormone is strongly correlated to the plant’s water status [50,51], and/or by the decreased photosynthetic efficiency. Abiotic stresses could compromise the dynamic *equilibrium* between intracellular generation and removal of ROS, whose over-accumulation may induce oxidative stress. In particular, excessive concentrations of radical species, such as H_2_O_2_, O_2_•^−^, and •OH, damage plant cell components, leading to cell death [8,9]. Investigations on the redox status of Sulla seedlings and plants were performed in this study, by assessing O_2_•^−^ and H_2_O_2_ levels. The amount of superoxide anion increased by 4-fold and 2-fold compared to the control in plants and seedlings exposed to salinity, respectively. Hydrogen peroxide accumulated exclusively in seedlings 2-fold with respect to the control. The enhancement of ROS production, due to abiotic stresses, generally affects both cellular and organelle membrane lipids by peroxidation, resulting in a loss of membrane integrity [52]. Lipid peroxidation is usually monitored via MDA, a degradation product of fatty acids. In Sulla samples, MDA content did not increase significantly with respect to the controls. Similarly, Bejaoui and colleagues [53] found that the MDA level in *S. coronaria* treated with 200 mM NaCl remained unchanged. These scholars suggested that Sulla may activate such a type of efficient antioxidant mechanism able to avoid lipid peroxidation, validating our results. Furthermore, Trypan Blue staining for the cell viability survey did not underline critical conditions in the samples. The increase of the ROS level not associated to phytotoxicity pointed out a potential function of these molecules as signal transduction messengers in plant tissues [54,55]. This evidence implied that ROS accumulation to a critical level was avoided by fine modulation of the antioxidative response. In fact, plants are known to possess a wide range of defence systems against the deleterious effects of oxygen radicals, including non-enzymatic and enzymatic antioxidants [5,48].

Phenolics and glutathione have been acknowledged as fundamental molecules in restoring cell redox balance, especially when oxidative stress occurs. Therefore, the increase of these compounds is generally linked to plant protective responses [56,57]. In this study, they were spectrophotometrically assayed. Total phenolic content increased exclusively in Sulla seedlings after the 200 mM NaCl treatment, revealing the possible involvement of these antiradical compounds in ROS scavenging for these samples. Since the activation of phenolic synthesis entails highly energetic metabolic pathways, the missed increase of phenolics in adult plants was likely due to energetic savings, consistent with the low photosynthesis efficiency registered in these specimens. Glutathione can act directly as a free radical scavenger or participate in AsA regeneration via the AsA-GSH cycle [58]. Total glutathione decreased in seedlings and remained at a steady state in plants, after drought and salinity treatments. GSSG/GSH ratios were measured to better determine the oxidative status of seedling and plant stressed tissues. The outcome revealed that the treatments induced a shift of the redox equilibrium towards oxidative conditions. Surprisingly, AsA levels did not change significantly after stress imposition, suggesting that this non-enzymatic antioxidant would not be particularly involved in the Sulla response to drought and salinity. As stated by Gill and colleagues [59], the AsA-GSH pathway includes several antioxidants, both enzymatic and non-enzymatic, that cooperate to protect cells against ROS accumulation. A future deeper investigation based on this specific pathway could surely provide a clearer picture of the role of these components in the response of Sulla to abiotic stresses. ROS enzymatic scavengers in plants include SOD, APX, and CAT; SODs convert anion superoxide into H_2_O_2_ and constitute the first line of defence, while CAT and APX decompose H_2_O_2_ into H_2_O [8]. In plants, three variants of SOD are known, differing for their subcellular localization and metal cofactor at the active site [14]. In the current paper, the activity of these three enzymes and the transcription level of their genes were investigated. After high salt exposure, SOD activity was lower in treated seedlings and plants with respect to the controls, thus explaining the high concentrations of superoxide we observed in these samples. By contrast, in plants exposed to drought, SOD function appeared unaltered. Consistently, mRNA abundance for the genes of SOD isoforms appeared lower or comparable to that of the controls. CAT activity seemed stable or decreased, coherently with the expression levels of its gene. In seedlings, the accumulation of H_2_O_2_ did not appear to be associated with upregulation of the SOD activity but probably to the incapacity of CAT and APX to degrade this radical species synthesized in different cell compartments (e.g., apoplast, chloroplasts, peroxisomes, mitochondria) [60]. Similarly, APXc transcripts were strongly reduced during the high salt stress, although its activity did not reveal significant changes. High salt-treated plants highlighted a decrease of the H_2_O_2_ level and an accumulation of O_2_•^−^; this phenomenon could be linked to the reduced activity of SOD. Consequently, the observed reduction of CAT gene expression and activity in the same samples was expected. By contrast, in plants exposed to drought conditions, the strong upregulation of CAT and APXc transcription was quite unforeseen because only a minimal decrease of H_2_O_2_ and no variation in APX and CAT activity was documented. Further studies should be performed to better interpretate this phenomenon and the divergence observed in some cases between activity and transcript amounts of the antioxidant enzymes. Indeed, it is well-known that the stability of mRNAs and the degradation rate of proteins are not always directly correlated. However, contradictory evidence related to APX finds support in the literature, which has documented that drought-stressed plants simultaneously showed overexpression of APX genes and unchanged enzyme activity [61,62], perhaps due to a wide and untargeted modification of the plant transcriptional pattern in response to abiotic stress. 

## 5. Conclusions

The principal intent of this study was to underline the dual role of the redox status in *S. coronaria*, both as a stress sensor and activator of the defence response, evaluating the effect that abiotic conditions have on the main oxo-reductive components, both in seedlings and plants. The present findings prove that the antioxidant system in Sulla is finely modulated by the applied abiotic stressors, suggesting its primary involvement in plant defence. In detail, ROS accumulation at a non-toxic level for plant tissues implied a possible role of these radicals as signal transduction molecules. Moreover, we observed that Sulla plants and seedlings differently acclimated themselves against salinity, maybe due to their distinctive genome plasticity and propensity to epigenetic modifications. All this evidence provides a new piece of information about the molecular mechanisms that underlie the response of *S. coronaria* to drought and salinity.

## Figures and Tables

**Table 1:** Biometric analysis: root length, hypocotyl length, and cotyledon length and width are reported for control and treated (100, 150, and 200 mM NaCl) seedlings of *Sulla coronaria* grown in agar. Results are expressed in cm, or mm, as means ± S.E. of eight different measurements (*n* = 8). The asterisks indicate the degree of significance with respect to the control (one-way ANOVA/LSD test): *** *p* < 0.001.

Treatment	Root Length (cm)	Hypocotyl Length (cm)	Cotyledon Width (mm)
Control	6.68 ± 0.33	2.03 ± 0.07	4.74 ± 0.15
100 mM	5.78 ± 0.42	1.85 ± 0.08	4.77 ± 0.15
150 mM	3.51 ± 0.51 ***	1.32 ± 0.12 ***	4.39 ± 0.16
200 mM	3.26 ± 0.47 ***	0.87 ± 0.11 ***	4.52 ± 0.17

**Table 2:** Photosynthetic pigment content (Chl *a*, Chl *b*, carotenoids) quantified in the leaves of plants and in whole seedlings of *Sulla coronaria* after exposure to three different regimes of water and salt stress. The values are means ± S.E. of three independent experiments (*n* = 3). The asterisks indicate the degree of significance with respect to the control samples (one-way ANOVA/LSD test): *** *p* < 0.001, ** *p* < 0.01, * *p* < 0.05.

Pigment (µg 100 mg^−1^ FW)			
Plants (drought)	Chl *a*	Chl *b*	Carotenoids
Control	81.58 ± 7.34	23.17 ± 2.40	19.95 ± 2.02
1 week	94.03 ± 2.02	22.69 ± 2.84	27.06 ± 1.27 *
2 weeks	103.93 ± 1.77 *	25.48 ± 1.77	28.57 ± 0.94 **
3 weeks	43.55 ± 8.54 **	10.86 ± 1.95 **	9.04 ± 2.30 **
Plants (NaCl)			
Control	81.58 ± 7.34	23.17 ± 2.40	19.95 ± 2.02
100 mM	31.05 ± 3.42 ***	7.61 ± 1.14 ***	7.05 ± 0.63 ***
150 mM	31.56 ± 0.18 **	7.63 ± 0.18 **	9.16 ± 0.07 *
200 mM	30.10 ± 1.29 ***	7.91 ± 0.12 ***	6.75 ± 0.62 ***
Seedlings (NaCl)			
Control	2.56 ± 0.46	1.46 ± 0.20	0.89 ± 0.12
100 mM	4.86 ± 0.54 **	1.98 ± 0.17	1.44 ± 0.13 **
150 mM	4.80 ± 0.74 *	1.80 ± 0.20	1.35 ± 0.19 *
200 mM	5.79 ± 0.67 ***	2.15 ± 0.20 *	1.58 ± 0.16 **

**Table 3:** Phenolic amount, total glutathione level, GSSG/GSH ratio, and ascorbic acid concentration in leaves of plants and whole seedlings of *Sulla coronaria* after three weeks of suboptimal irrigation and after treatment with 200 mM NaCl. Data are means ± S.E. of three different measurements (*n* = 3). The asterisks denote significant differences from control samples (one-way ANOVA/LSD test; *** *p* < 0.001, ** *p* < 0.01, * *p* < 0.05).

Phenolics (μg GAE 100 mg^−1^ FW)		
Plants		
Control	30.80 ± 5.09	
3 weeks drought	38.21 ± 9.88	
200 mM NaCl	12.96 ± 1.96 **	
Seedlings		
Control	19.73 ± 1.50	
200 mM NaCl	45.91 ± 4.85 ***	
**Glutathione (μM 50 mg^−1^ FW)**		
	Total glutathione	GSSG/GSH
Plants		
Control	137.56 ± 14.49	0.21
3 weeks drought	140.28 ± 13.05	0.66 *
200 mM NaCl	130.16 ± 9.68	0.80 *
Seedlings		
Control	156.09 ± 12.13	0.14
200 mM NaCl	96.38 ± 6.31 **	0.63 **
**Ascorbic acid (** **μ** **g g^−1^ FW)**		
Plants		
Control	36.7 ± 1.55	
3 weeks drought	41.2 ± 2.12	
200 mM NaCl	43.7 ± 2.33	
Seedlings		
Control	30.2 ± 1.52	
200 mM NaCl	27.9 ± 1.32	

## Data Availability

The data supporting the findings of this study are reported in the paper, in its supplementary data or available from the corresponding author, Angelo Gismondi, upon request.

## Supplementary Materials

The following are available online at https://www.mdpi.com/article/10.3390/antiox10071048/s1. Table S1. Primers used in qRT-PCR. Figure S1. *Sulla coronaria* plants before stress imposition. Figure S2. Water content of *Sulla coronaria* plants and seedlings after exposure to drought and salinity. Figure S3. Trypan blue staining for the detection of dead cells in *Sulla coronaria*. Figure S4. Controls for DCF assay.
