# Supplementary material for: Investigating the Drought and Salinity Effect on the Redox Components of Sulla Coronaria (L.) Medik
Source: Antioxidants (Basel). 2021 Jun 29;10(7):1048. doi: 10.3390/antiox10071048 (PMC8300714; doi:10.3390/antiox10071048)

Supplementary Materials: Investigating the Drought and Salinity Effect on the Redox Components of *Sulla coronaria* (L.) Medik.

Silvia De Rossi [1,2], Gabriele Di Marco [1], Laura Bruno [1], Angelo Gismondi [1,*,†] and Antonella Canini [1,†]

1 Department of Biology, University of Rome "Tor Vergata", 00133 Rome, Italy; silvia.derossi@alumni.uniroma2.eu (S.D.R.); gabriele.di.marco@uniroma2.it (G.D.M.); laura.bruno@uniroma2.it (L.B.); gismondi@scienze.uniroma2.it (A.G.); canini@uniroma2.it (A.C.)
2 PhD Program in Evolutionary Biology and Ecology, Department of Biology, University of Rome "Tor Vergata", 00133 Rome, Italy
* Correspondence: gismondi@scienze.uniroma2.it
† A.G. and A.C. are co-last authors of the present work.

Table S1. Primers used in qRT-PCR.

Fig. S1. *Sulla coronaria* plants before stress imposition.

Fig. S2. Water content of *Sulla coronaria* plants and seedlings after exposure to drought and salinity.

Fig. S3. Trypan blue staining for the detection of dead cells in *Sulla coronaria*.

Fig. S4. Controls for DCF assay.

Table S1. Primers used for the quantitation of mRNA levels by qRT-PCR.

Gene	Sequence*	
CuZnSODc	F:	5'-TGAACAATGGTGAAGGCTGTG-3';
	R:	5'-CCTTGACATTATCGCTGCTGC-3'
FeSODp	F:	5'-TCGCCATCCACTTCCACTACT-3';
	R:	5'-GAAACGCGGAAGGTGATGAT-3'
MnSOD	F:	5'-CCTCAGCCGTCGTTAAGCTC-3';
	R:	5'-GACCTCCGCCATTGAATTTG-3'
APXc	F:	5'-AAGGCCAGGAGAAAGCTCAGA-3';
	R:	5'-AGCAGAGTGCCATGCCAAA-3'
CAT	F:	5'-GCGCCTGACAGGCAAGATA-3';
	R:	5'-TGCCCAAGAGAACGATCAGC-3'

* Rubio, M.C.; Bustos-Sanmamed, P.; Clemente, M.R.; Becana, M. Effects of salt stress on the expression of antioxidant genes and proteins in the model legume *Lotus japonicus*. *New Phytol.* **2009**, *181*, 851–859.

Figure S1. *Sulla coronaria* plants (45-days old) grown in pots before stress imposition for *in vivo* experiments.

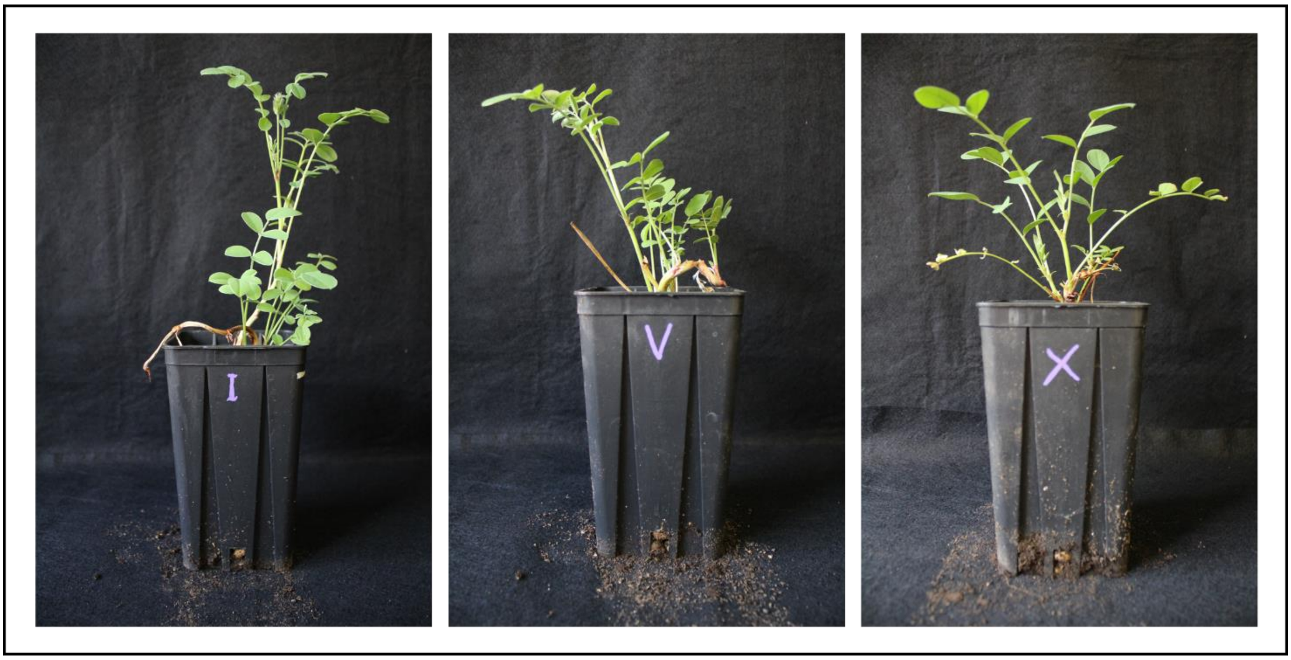

Figure S2. Water content (WC) of whole plants (A) and whole seedlings (B) of *Sulla coronaria*, after exposure to three different regimes of drought and salinity. Results are reported as percentage and represent means ± S.E. of three different replicates (n=3). No statistically significant differences were observed (one-way ANOVA/LSD test).

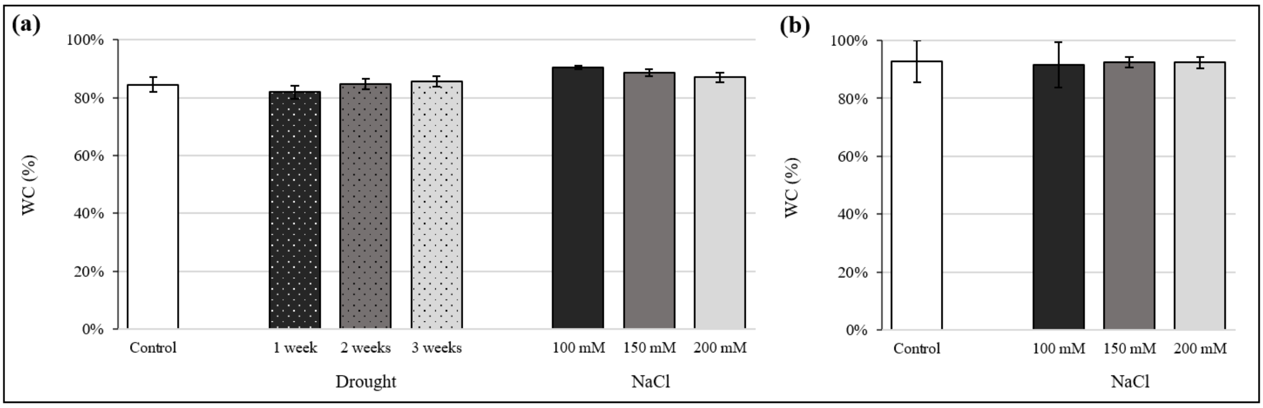

Figure S3. Trypan blue staining for the detection of dead cells in *Sulla coronaria*. (**a**) Leaves of plants after exposure to three levels of water stress; (**b**) effect of three different NaCl concentrations on leaves from plants; (**c**) effect of the same salt concentrations on cotyledons from seedlings. Necrotized leaf (or cotyledon) area was measured and compared with the total surface of the corresponding leaf

(or cotyledon) by Fiji/ImageJ software. The experiment was repeated three times (n=3). No significance difference among treatments and controls was registered (one-way ANOVA/LSD test).

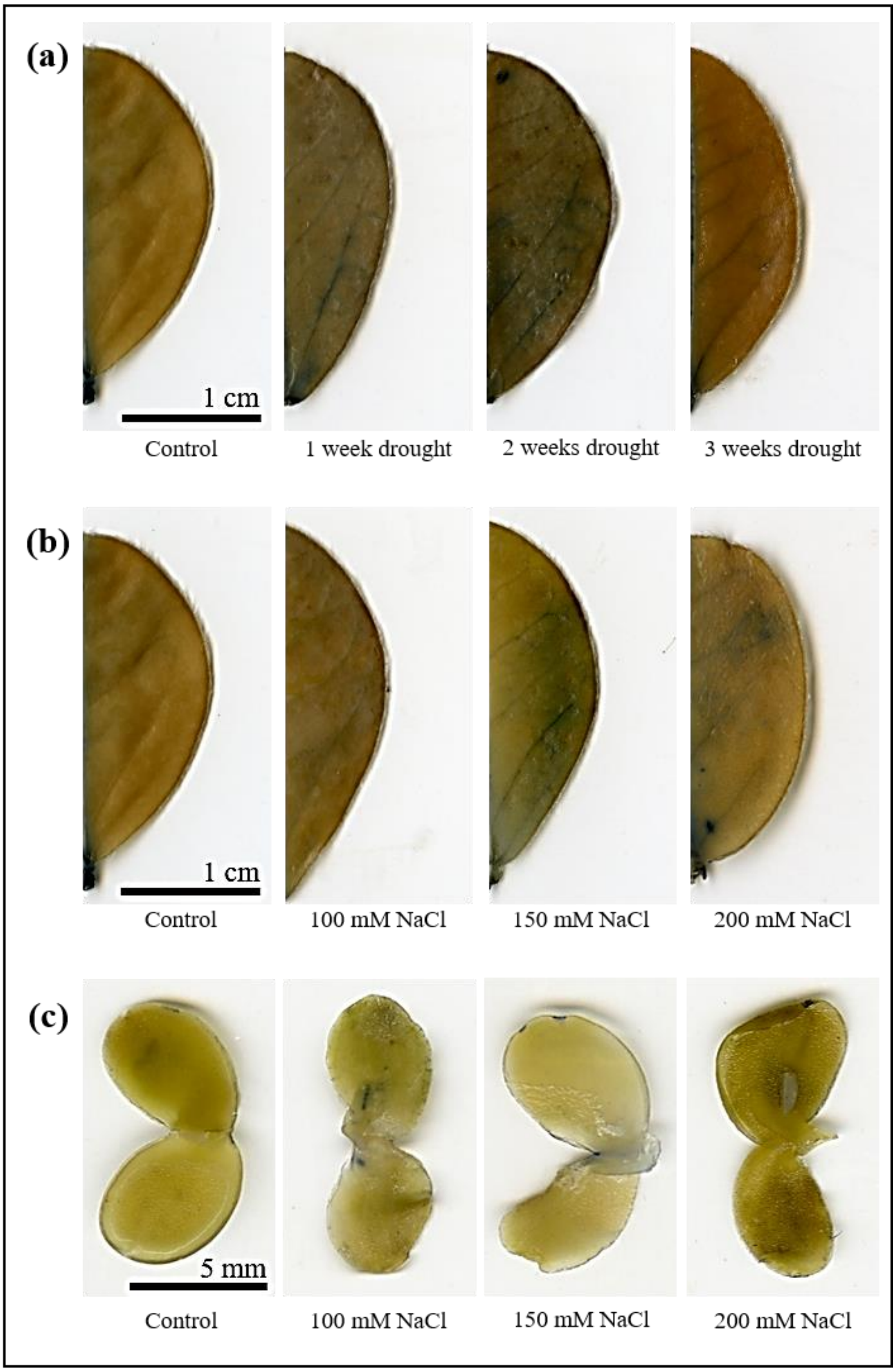

Figure S4. Negative and positive controls were carried out treating samples, for 1 h in agitation at 37 °C, with 1 mM of ascorbate and 5 mM H_2O_2, respectively. The bar indicates 20 μm.

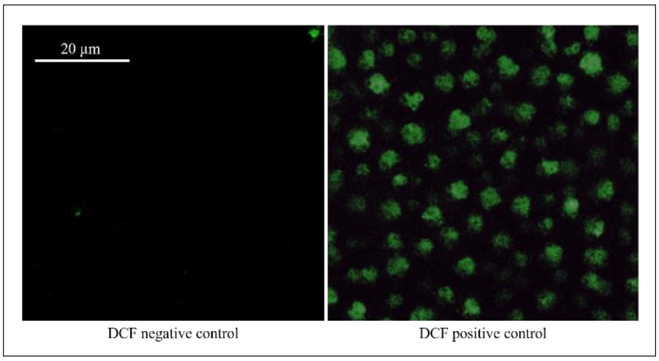

Supplement: Supplementary file 1 [file antioxidants-10-01048-s001.zip › antioxidants-1264831-supplementary.pdf]
